# Enteropathogens: Tuning Their Gene Expression for Hassle-Free Survival

**DOI:** 10.3389/fmicb.2018.03303

**Published:** 2019-01-09

**Authors:** Ritika Chatterjee, Meghanashree M. Shreenivas, Rohith Sunil, Dipshikha Chakravortty

**Affiliations:** ^1^Department of Microbiology and Cell Biology, Indian Institute of Science, Bengaluru, India; ^2^Division of Biological Sciences, Indian Institute of Science, Bengaluru, India; ^3^Undergraduate Studies, Indian Institute of Science, Bengaluru, India; ^4^Centre for Biosystems Science and Engineering, Indian Institute of Science, Bengaluru, India

**Keywords:** enteropathogen, quorum sensing, *E. coli*, Shigella, Salmonella

## Abstract

Enteropathogenic bacteria have been the cause of the majority of foodborne illnesses. Much of the research has been focused on elucidating the mechanisms by which these pathogens evade the host immune system. One of the ways in which they achieve the successful establishment of a niche in the gut microenvironment and survive is by a chain of elegantly regulated gene expression patterns. Studies have shown that this process is very elaborate and is also regulated by several factors. Pathogens like, enteropathogenic *Escherichia coli* (EPEC), *Salmonella* Typhimurium, *Shigella* *flexneri*, *Yersinia* sp. have been seen to employ various regulated gene expression strategies. These include toxin-antitoxin systems, quorum sensing systems, expression controlled by nucleoid-associated proteins (NAPs), several regulons and operons specific to these pathogens. In the following review, we have tried to discuss the common gene regulatory systems of enteropathogenic bacteria as well as pathogen-specific regulatory mechanisms.

## Introduction

Bacteria with their supposedly primitive genomic architecture, have proven to be one of the smartest living things to have existed. Their success of survival depends on a chain of elegantly regulated gene expression patterns. This regulation becomes all the more important in the case of pathogens, which have to cleverly sabotage the host immune responses. For successful entry and survival in the host of an enteric pathogen, these bacteria have to cross the acidic pH of the stomach, find a way to cross the barrier of the existing intestinal microbiota, escape from the cationic anti-microbial peptides and immunoglobulins in the intestine. After breaching all of the above-mentioned barriers and entering a cell, survival becomes harder with the acidification of phagosomes, burst of reactive oxygen and reactive nitrogen species, iron deprivation by the cells, and autophagy. Enteropathogenic bacteria have been the cause of most of the foodborne illnesses. Diseases ranging from diarrhea to systemic fever like typhoid caused by the *Salmonella* Typhi to life-threatening sepsis are caused by these pathogens. In 2015, the World Health Organization (WHO) has reported an estimate of 582 million cases of 22 different foodborne enteric diseases and 351,000 associated deaths. Among these, 520,00 deaths were because of *Salmonella* Typhi and 370,00 deaths because of enteropathogenic *Escherichia coli* (EPEC). The disease burden is very high in Africa followed by southeastern Asia. Forty percent of the disease is reported in children under 5 years of age.

Pathogenesis of bacteria like EPEC and *Salmonella* Typhi have been fairly well characterized. However, with the emergence of multiple drug-resistant strains and the acquisition of many virulence genes by the bacteria with the horizontal gene transfer (HGT), control of disease progression has been difficult. Thorough understanding of the components dictating the life of these bacteria, inside and outside of the host is thus, vital. The following review discusses the gene regulation of some of the major enteric pathogens and some of the common themes of gene regulation in these bacteria.

## Gene Regulatory Systems

### Regulation of Gene Expression by Toxin- Antitoxin (TA) Systems

The success of the pathogenic bacteria has been heavily reliant on the retention of virulence factors in mobile genetic elements (Cambray et al., 2010) like pathogenicity islands (PAI), virulence plasmids and conjugative integrons. The existence of such TA cascades require the continuous generation of the antitoxin molecule for their survival and thus help in the retention of the plasmid. There have been ever increasing roles of these TA modules emerging in bacterial pathogenesis.

Toxins are generally protein molecules and antitoxins are protein/RNA molecules that sequester the toxins. Induction of TA modules occur with the RNase or protease mediated antitoxin degradation of antitoxin molecule. This results in the release of toxin that kills the bacteria. TA modules have been classified into six types, depending on the mechanism by which the antitoxin inhibits the toxin activity (Page and Peti, 2016). Type I consists of the TA systems where the antitoxin is the antisense RNA that binds to the toxin mRNA and this heteroduplex formation inhibits translation of the toxin. Type II consists of the TA systems where antitoxin is also a protein molecule which forms a tight complex with the toxin, hiding its active site, thus resulting in inhibition. Type III consists of the TA systems where the antitoxin is a small RNA molecule that directly interacts with the toxin and inhibits activity. Type IV has TA systems in which the antitoxin protein molecule directly binds to the target of the toxin, thus inhibiting the toxin activity. TA systems in which the antitoxin molecule acts as a ribonuclease specific to the toxin mRNA constitute Type V and Type VI has the TA systems wherein the antitoxin brings about the proteolytic degradation of the toxin. Enteropathogenic bacteria have acquired many of these TA systems which reside in the virulence plasmids and horizontally acquired PAIs. Following are some of the TA systems existing in enteropathogenic bacteria which aid in their survival.

*Vibrio cholerae* have seven *relBE* Type II TA systems which are induced upon stress conditions experienced by the bacteria in the host. RelE is the toxin then cleaves mRNA leading to the inhibition of growth whose activity is inhibited in the presence of RelB antitoxin. Deletion of this TA system has been shown to hamper the ability of the pathogen to both survive and colonize in the host (Wang et al., 2015). Out of all the enteropathogenic bacteria, *Salmonella* species have the most number of well characterized TA systems. It possesses six putative Type I TA modules and seventeen Type II TA systems (Lobato-Marquez et al., 2015). One such Type II TA module present in one of the virulence plasmids, VapBC2ST helps the survival of *Salmonella* Typhimurium inside the host fibroblasts and epithelial cells (Lobato-Marquez et al., 2015). VapC being the toxin acts as an RNAse that cleaves the mRNA transcript independent of the ribosomes. Since the activation is cell type specific, activity of this particular TA system might be to dictate the outcome of the pathogenesis. This is highly homologous to *mvpAT* TA system located on the virulence plasmid of *Shigella* *flexneri* which is essential for the maintenance of the plasmid (Sayeed et al., 2005). Recent reports have also shown that positioning this TA system near to the 30 kb PAI leads to both global and local gene regulation. This also leads to the loss of expression of the Type III secretion system by the plasmid. Thus, altering the ability of the pathogen to invade (Pilla et al., 2017).

Another Type II TA system, SehAB is found to be important during the initial phases of infection of *Salmonella*. Especially in the survival of the pathogen in the mesenteric lymph nodes. This was demonstrated with the comparison of survival of a wild-type STM strain and a strain lacking SehAB. The mutant strains exhibited decreased virulence as compared to the wild-type when administered through the oral route whereas the virulence was not hindered when administered through intra-peritoneal route (De la Cruz et al., 2013). SehA, the toxin is the homolog of HigB, which is a ribosome-dependent endoribonuclease. In conditions that are known to increase the expression of genes involved in the intracellular proliferation, SehAB transcription is found to increase. Although SehAB transcription is found to be increased upon infection of the macrophages, it is not required for the replication. It is also not required for replication in bone marrow-derived macrophages or in epithelial cells like HeLa (Lobato-Marquez et al., 2015).

Toxin RelE was found to be important in the virulence as mutants that lacked RelE like toxin showed reduced virulence as compared to the wild-type. RelE toxin either directly or indirectly cleaves the mRNAs associated with the ribosomes. Toxins such as T4ST, T5ST, T2ST are found to be differentially expressed in fibroblasts as compared to the epithelial cells. Transcription of at least one of the three type I TA systems, namely, *tisB-istRST*, *hok-sokST*, *idrA-rdlAST* and two of the type II TAs, *ta4ST* and *vapBC2ST*, are found to be indispensable for the survival of STM inside fibroblasts (Lobato-Marquez et al., 2015). It is also observed that the type I and the type II TA systems differentially modulate survival in fibroblasts and epithelial cells. Thus, the existence of extrachromosomal genetic elements, harboring TA systems, play vital roles in the virulence of the enteropathogenic bacteria (Table 1).

**Table 1:** Summarizing some of the TA systems essential for virulence of the enteric pathogens.

Bacteria	TA systems	Type I	Function	Reference
*Salmonella* Typhimurium	tisB-istR_ST_, hok-sok_ST_, idrA-rdlA_ST_		Survival in fibroblasts	Lobato-Marquez et al., 2005
*Salmonella* Typhimurium	SehAB	II	Essential for initial survival in MLN	De la Cruz, 2013
*Salmonella* Typhimurium	VapBC2_ST_	II	Survival in epithelial cells and fibroblasts	Lobato-Marquez et al., 2005
*Salmonella* Typhimurium	Ta4_ST_	II	Survival in epithelial cells	Lobato-Marquez et al., 2005
*Salmonella* Typhimurium	tisB-istR_ST_, hok-sok_ST_, idrA-rdlA_ST_	I	Survival in fibroblasts	Lobato-Marquez et al., 2005
*Shigella flexneri*	MvpAT	II	Survival in epithelial cells	Sayeed et al., 2005
*Vibrio cholerae*	RelBE	II	Survival and colonization in host	Wang et al., 2015


Type IV TA system *yeeUV* in *E. coli* inhibits cell division. The antitoxin molecule YeeU helps in the polymerization of MerB and FtsZ by allowing them to bundle and thus protecting them from the action of the toxin YeeV (Masuda et al., 2012). The only reported Type V TA system is the *ghoST* system. GhoT is the toxin whose mRNA ids cleaved by the antitoxin GhoS. GhoT disrupts the membrane potential and thus, ATP synthesis (Wang et al., 2012). Interestingly, the *ghoST* TA system is recently found to be regulated by another TA system, *mqsRA* (Wang et al., 2013). MqsR toxin cleaves the *ghoS* transcript and thus, there is also cross regulation found among the existing TA systems.

The number of TA systems characterized to be exhibiting specific functions is miniscule when compared to the number of TA systems reported. Research devoted to characterization and a better understanding of these TA systems would open a wide arena for research wherein the TA systems can be employed to trick bacteria to negatively regulate its own virulence genes, as was done with the *mvpAT* of *Shigella*.

The induction of certain TA systems is also quorum dependent. One such chromosomal Type II TA system found in *E. coli* is *mazEF* which is regulated in a quorum dependent manner. The induction is brought about by the degradation of the antitoxin MazE by the Lon protease or other proteases depending on the stage of growth of the bacteria (Aizenman et al., 1996; Christensen et al., 2003). In the following section, we will see a more detailed quorum dependent gene regulatory systems.

### Regulation of Gene Expression by Quorum Sensing

Quorum sensing (QS) represents the ability of bacteria to sense the population density in the surrounding environment. The way it does is through cell-to-cell signaling mechanism, by producing and/or responding to a chemical signal called autoinducer (AI). Gene expression is regulated depending on the surrounding concentration of AI. More than 45 years ago this phenomenon was discovered in the regulation of bioluminescence of *Vibrio harveyi* (Nealson et al., 1970) and later in *Vibrio fischeri* (Hastings and Nealson, 1977). One such diverse environment for bacterial crosstalk and signaling between beneficial microbiota, invading pathogen and the mammalian host is the large intestinal milieu. Inside intestinal milieu, beneficial microbiota helps the host in nutrient assimilation and immune competence (Hooper and Gordon, 2001). Pathogenic or opportunistic organisms can also occasionally establish their niche and thereby manifest a disease condition. QS is crucial because of the high density of microbes in the large intestine where interspecies, intraspecies and cross-kingdom coordination must be established. The enteric pathogens use mainly three types of QS systems: Lux R-I, LuxS/AI-2 and AI-3/epinephrine/norepinephrine. A thorough review of the kind of QS systems present and utilization of the same by pathogenic as well as commensal bacteria has been provided here. It will also shed some light on the kind of approach one can take to limit food borne pathogens disease manifestations.

### Lux R-I

The regulation of bioluminescence in *Vibrio* sp. is through Lux R-I system. There are two regulatory proteins: Lux I, which is responsible for the production of *N*-acyl-homoserine-lactone (AHL) AI and Lux R, which get activated by AI thereby increasing transcription of the luciferase operon (Engebrecht et al., 1983; Engebrecht and Silverman, 1984). A similar regulation system has been discovered in gut-pathogen bacteria where homologs of LuxR-I operate and only difference lies in the downstream target gene regulation (de Kievit and Iglewski, 2000). *Yersinia* species also have LuxI and LuxR homologs including *Yersinia enterocolitica* which causes yersiniosis harbors only one pair of LuxRI (YenRI) in contrast to other species of *Yersinia* which harbor two pairs. *yenI* is involved in the synthesis of two short chains AHLs, along with three long-chain AHLs (Atkinson et al., 2006). One of the initial reports on *Yersinia enterocolitica* suggests that LuxR-I QS is involved in regulating the expression of the flagellar structural gene, *fleB* which in turn governs swimming motility (Atkinson et al., 2006). There are certain hypervirulent strain harboring a putative orphan *luxR* gene which is not linked to AHL synthase, *ycoR*. This QS system has been shown to regulate host cell attachment along with virulent plasmid *pYve* maintenance (Ng et al., 2018). *Y. enterocolitica* utilizes the type III secretion system (T3SS) to deliver multiple *Yop* effector proteins. It is regulated by Ca^2+^ ion concentration and temperature thereby maintain a tight control of phenotype (Cornelis et al., 1998; Cornelis, 2002; Trosky et al., 2008; Dewoody et al., 2013). This could probably improve the infectivity of the pathogen in terms of energy conservation and quick switch from not expressing virulent genes to expressing the same. *Y. pseudotuberculosis* and *Y. pestis* have convergently expressed two pairs of *luxI/R* orthologs. All the four major AHLs are produced by both the species.

On the other hand, *E. coli*, *Salmonella* and *Shigella* lack *luxI* gene, therefore, can’t synthesize AHLs. However, these organisms can respond to AHLs produced by other bacteria, with a receptor-like protein (SdiA). SdiA has an amino acid sequence homology with LuxR -type transcriptional factor (Yao et al., 2006). Yao et al. (2006) shows that the presence of several AHLs compounds is required for proper folding of SdiA. SdiA in *E. coli* K12 and EHEC was involved in cell division, increased quinolone resistance (Rahmati et al., 2002) and expression of virulence gene but only when SdiA is expressed in a high copy number plasmid. Chromosomally expressed SdiA regulate four genes involved in glutamate-dependent acid resistance and downregulate *fliE* in *E. coli* k12 and EHEC. Dyszel et al. (2010b) have thus concluded that gene regulation by SdiA in *E. coli* is only partially dependent on AHLs. The regulation through SdiA is only observed at 30°C also acid tolerance in *E. coli* is conferred by the same (Van Houdt et al., 2006). In *E. coli*, AHLs regulate adhesion to epithelial cells (Hep-2), biofilm formation (Lee et al., 2009) and acid tolerance (Van Houdt et al., 2006; Sharma and Bearson, 2013).

Inceptive studies on *Salmonella* have shown that SdiA was involved in regulation of exclusively one gene *rck* which functions to impart resistance against human complement. The SdiA in *Salmonella* is induced at low pH (pH 4) during aerobic conditions. Although SdiA mutant, can survive in Luria-Bertani medium minimum for a week at pH 4 (Rychlik and Barrow, 2005). The SdiA of *Salmonella* Typhimurium was activated during transit through the gastrointestinal tract of turtle colonized with *Aeromonas* *hydrophila*, capable of AHL synthesis (Smith et al., 2008). Also, report from Dyszel et al. (2010a)shows that *Salmonella* Typhimurium SdiA was activated during the transit through mice gut colonized by *Yersinia enterocolitica*, which is also capable of synthesis of AHLs. In the same study, they have shown that cloning *luxI* of *Yersinia* into *Salmonella* gave a benefit of fitness to the pathogen in mice model (Dyszel et al., 2010a), these studies suggest that even though *Salmonella* lacks AHL producing capabilities it can very well respond to it via orphan receptors such as SdiA. This will not only help *Salmonella* to crosstalk but could be one of the mechanisms to establish co-infection.

In *Salmonella*, the AHLs also regulate survival in rabbit and guinea pig serum apart from anchoring and invasion in HeLa and HEp-2 cells respectively (Nesse et al., 2011; Liu et al., 2014; Campos-Galvao et al., 2016). During micro-aerobiosis and anaerobiosis conditions the gene expression through LuxS and SdiA is also reduced (Lamas et al., 2016). Therefore, it is evident that entero-pathogens regulate their virulence factors at transcriptional level itself via LuxRI systems. These virulence factors are majorly associated with establishment of infection. This often serves them with an upper hand in a competitive environment such as intestinal milieu.

### LuxS/AI-2

Most of the sequenced bacterial genomes have *luxS* QS system (Waters and Bassler, 2005) and hence it is a universal signaling molecule which serves the purpose of interspecies communication. Subsequently, it was found that the enzyme *luxS* which is responsible for AI-2 synthesis is extremely conserved and globally expressed among a variety of bacterial species (Han and Lu, 2009; Pereira et al., 2013; Even-Tov et al., 2016) Primarily it was identified in *V. harveyi* for regulation of bioluminescence (Surette et al., 1999). The key enzyme in this sensing is LuxS which has a role in *S*-adenosyl methionine metabolism. LuxS catalyzes the reaction of *S*-ribosyl-homocysteine into homocysteine and highly unstable 4,5- Dihydroxy 2,3 pentanedione, which cyclizes to form furanones (Schauder et al., 2001; Winzer et al., 2002a; Sperandio et al., 2003), serving as a precursor of AI-2 compound(Schauder et al., 2001). AI-2 is imported inside the cell by specific receptor transport system present on bacterial membrane. Several previous works have identified various AI-2 receptors in diverse bacterial species. LuxP was discovered in *V. harveyi*, LsrB in *Samonella* Typhimurium, and RbsB in *Haemophilus influenzae* (Armbruster et al., 2011). The LuxS/AI-2 type of QS system has been found to be involved in the regulation of bacterial bioluminescence, competence, biofilm formation, antibiotic resistance (Xue et al., 2013), sporulation, and virulence factor secretion (Novotny et al., 2015), type three secretion system (Han et al., 2013, 2015a,b). In *Salmonella*, it was initially reported that AI-2 only regulated gene expression of ABC transporter named Lsr (LuxS regulated). AI-2 is a transcriptional activator of *lsr* operon which consists of seven genes (*lsrACDBFGE*) (Taga et al., 2001). The Lsr transporter was also reported in *E.* *coli* and in both bacteria have homology with sugar transporter. Once AI-2 is inside the cell it is further phosphorylated thereby interacts with LsrR which is a SorC-like transcription factor that represses *lsr* operon (Taga et al., 2001; Taga et al., 2003). The homology between *lsr* transporter and sugar importer lead to suggesting that the function of AI-2 is basically metabolic (Winzer et al., 2002b, 2003; Vendeville et al., 2005) The AI-2 import is tightly regulated and remains shut off in presence of glucose because of repression mediated by cAMP-catabolite activator protein (CAP) (Wang et al., 2005; Xavier and Bassler, 2005). Microarray based study has shown that *luxS* mutation leads to multiple metabolic changes, especially in processes involving nitrogen and carbon metabolism (Walters et al., 2006). AI-2 mediated signaling has also been shown to modulate the gut microbiota to facilitate colonization of a Firmicutes while hindering the Bacteroidetes during antibiotic mediated dysbiosis (Thompson et al., 2015). It is also reported for identification of AI-2 receptor in recombinant *E*. *coli* background. AI-2 interacts with LsrB in a temperature dependent manner (Zhang et al., 2017). Recent work on S. Dublin also shows that AI-2 is involved in biofilm formation and antibiotic resistance (Ju et al., 2018). Even though the genetic regulation via AI-2 system majorly involves metabolic control and interspecies crosstalk, there is some host protective role of commensals via AI-2 signaling to maintain the quorum of enteric microbiota. Therefore, AI-2 system can be said as a two-way sword which helps the host and can be also utilized by pathogens to suppress the inhibition by commensals to establish successful infection in host.

### AI-3/Cross-Kingdom Quorum Sensing

The third and most diverse type QS involves with AI-3 compound which facilitates cross-kingdom signaling between interspecies and inter-kingdom (host–microbes). Mammalian host produces a variety of hormones to maintain it’s signaling these include peptide and steroidal hormones. These hormones are then sensed by microflora residing in our system and serves as a cue for modulating their gene expression. The AI-3 compound is different from AI-2 and the former is capable of binding C-18 column and elution is possible only with methanol (Sperandio et al., 2003). The AI-3 is involved in quorum-sensing mechanisms to regulate the genes associated with EHEC LEE, flagellar and motility (Sperandio et al., 1999, 2002). AI-3 is produced by commensal organisms of gut microflora or by the host (epinephrine/norepinephrine). Mass spectroscopy analysis has shown that there are structural similarities between AI-3 and host epinephrine/norepinephrine, therefore, it is hypothesized as an antagonist of adrenergic receptors would also block QS through AI-3(Sperandio et al., 2003). During EHEC infection, the host adrenaline/ noradrenaline (NA) can’t cross the cellular membrane, thus it employs a two-component systems (TCSs) QseBC and QseEF to carry the signal inside bacteria. The two-component system has a membrane-bound histidine kinase which senses the signal and phosphorylates downstream cytoplasmic response regulator (RR) that results in the regulation of target genes (Jung et al., 2012). In the case of EHEC, genes regulated by QseC via adrenaline and NA are most often associated with flagella (Clarke and Sperandio, 2005b), motility (Reading et al., 2009), stress responses, ion uptake, osmolarity regulation, attaching and effacing lesions formation on enterocytes (Nakashima et al., 1992; Sperandio et al., 2002; Clarke and Sperandio, 2005a; Reading et al., 2007; Hughes et al., 2009; Njoroge et al., 2012). QseC is indispensable for EHEC virulence, and deletion leads to attenuation in rabbit and bovine models. QseEF regulates the gene which is involved in Shiga toxins synthesis, SOS responses, and other TCSs, which includes RcsBC, PhoPQ (Reading et al., 2009, 2010; Njoroge and Sperandio, 2012). Similarly, other hosts signaling molecules are also sensed by gut microflora which includes peptide hormones such as gastrin (Chowers et al., 1999), EGF, natriuretic peptides (Veron et al., 2007; Blier et al., 2011) and opioid hormones (Beury-Cirou et al., 2013).

Apart from these host-microbe interaction bacteria have also ability to sense host active defense/immune components such as cytokines (Porat et al., 1991; Luo et al., 1993; Zav’yalov et al., 1995; Wu et al., 2005), antimicrobial peptides (Miller et al., 1989; Bader et al., 2005) along with various host membrane component such as phosphatidylethanolamine (Bertin et al., 2011; Thiennimitr et al., 2011; Kendall et al., 2012; Luzader et al., 2013; Gonyar and Kendall, 2014), sugars (Pacheco et al., 2012) and host antioxidants, glutathione (Reniere et al., 2015; Kendall and Sperandio, 2016). Sensing the host intestinal environment and counter responding to the same is one of the preliminary strategies to evade host immune system and establishing the infection. AI-3 QS being the major crosstalk between host–pathogen rather helps the pathogen to comprehend the host anti-pathogenic retaliations.

**FIGURE 1:**
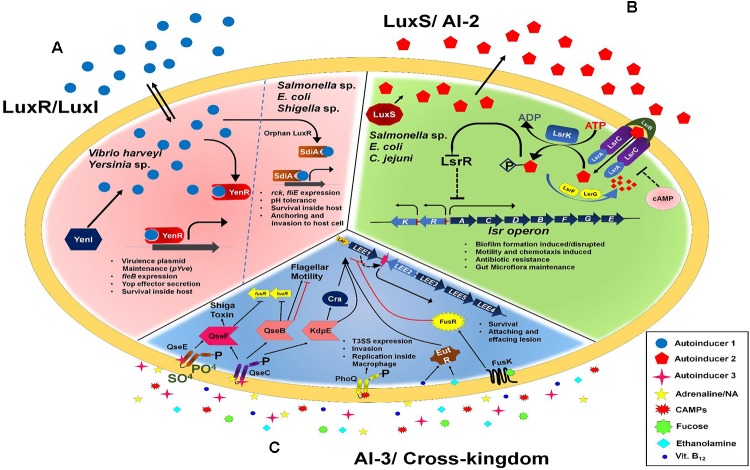
Quorum sensing in an overview, **(A)** describes the AI-1 mediated Quorum sensing and its downstream target gene regulation, **(B)** describes the AI-2 mediated gene regulation through *lsr* operon, **(C)** Cross-kingdom communication to facilitate the bacteria to sense the cues from the host and thereby establish successful infection.

There are several other enteric bacterial genera such as *Shigella*, *Salmonella*, Klebsiella, Enterobacter, and Citrobacter which produces the AI-3 compound and are used for inter-species communication (Walters et al., 2006). In enteric bacteria genera *Shigella*, *Salmonella*, and *Yersinia* it has been reported to have amino acid sequence conservation for QseC sensors (Clarke et al., 2006). Combining all the type of QS system we have tried to summarize in schematic (Figure 1). Targeting these molecules could be one of the smart ways for future therapeutics for food and water-borne diseases.

### Regulation of Gene Expression by Nucleoid-Associated Proteins (NAPs)

Nucleoid-associated proteins are proteins in non-eukaryotes involved in the compact storage of genetic material. They are responsible for altering the direction of DNA in the nucleoid, i.e., the configuration of the DNA in three-dimensional space by bending, wrapping and bridging it. They are known to regulate gene expression at the transcriptional-level (Dillon and Dorman, 2010).

Actively growing bacteria are probably shown more stable looped chromosomal domains in line with higher transcriptional activity such as synthesis of rRNA and other structures involved in translation (Postow et al., 2004; Deng et al., 2005; Stein et al., 2005). NAPs such as H-NS and factor of inversion (Fis), are found to bind to genomes (Grainger et al., 2006; Lucchini et al., 2006; Navarre et al., 2006; Noom et al., 2007). The binding locations of H-NS are believed to function as chromosomal domain loop boundaries (Noom et al., 2007). Proteins that bridge DNA are conjectured to be the best candidates for the formation of domain loop boundaries (Dillon and Dorman, 2010). Interestingly, nucleoid structure is found to be maintained even in the absence of Fis and H-NS among other NAPs (Zimmerman, 2006).

DNA-protein-DNA bridges are believed to be capable of either enabling or disabling the RNA polymerase to bind to DNA (Dame et al., 2002; Grainger et al., 2006). StpA and Sfh are H-NS paralogs and can form heteromers with H-NS (Dorman, 2004). The change in the numbers of the multimeric NAPs is shown to change gene expression patterns (Muller et al., 2006). Haemolysin expression modulating (Hha)/YdgT protein’s interaction with H-NS influences gene regulation activity of H-NS (Madrid et al., 2007). H-NS also plays a key role in the regulation of certain virulence genes in response to temperature (Rowe et al., 2000).

HU is another NAP. It has two sub-units, Huα and Huβ. In *E. coli* HU exists as either heterodimer or homodimer according to the stage of growth (Claret and Rouviere-Yaniv, 1997). HU influences the expression of a broad spectrum of genes in *E. coli* which includes genes involved in central metabolism and respiration (Oberto et al., 2009). It interacts with topoisomerase I and induces changes in the super-helicity of the DNA, thereby influencing gene expression (Broyles and Pettijohn, 1986). In several instances, the activity of promoters can be increased or decreased in response to super-helical variation (Dorman, 2006). HU also induces DNA bending which enables the formation of DNA loops which influences gene regulation and at the same time H-NS has the opposite effect (Becker et al., 2007).

Unlike HU, IHF binds to a well-conserved nucleotide sequence eventually causing the DNA to take a U-turn centered at the binding site of the protein to the DNA (Swinger and Rice, 2004; Dillon and Dorman, 2010). In *E. coli* and its relatives, IHF consists of an alpha subunit and a beta subunit. The alpha-beta heterodimer is the predominant active form, but it has been shown that the alpha–alpha and beta–beta homodimers are also biologically active in *Salmonella* *enterica* subspecies enterica serovar Typhimurium (Mangan et al., 2006). IHF has an influence on the global transcription in *E.* *coli* (Arfin et al., 2000) and *Salmonella* Typhimurium (Mangan et al., 2006). IHF recruits ^σ^54-containing RNA polymerase to promoters (Macchi et al., 2003). The *ilvPG* promoter of the *ilvGMEDA* operon of *E.* *coli* is activated by IHF through the release of free energy that is normally stored in the IHF-binding site, making this energy available to assist with the formation of the open transcription complex at the promoter (Sheridan et al., 1998).

Besides the mentioned NAPs there are several more with functions involved in the structuring of the DNA, gene expression and DNA replication. Being directly associated with the DNA give these set of proteins a direct advantage and a major chance of being involved in the regulation of gene expression.

Now that we have summarized some of the broad gene regulatory mechanisms that the enteropathogenic bacteria employ, we will discuss some of the pathogen specific mechanisms.

## Enteric Pathogens

### Gene Regulation in *Escherichia coli*

*Escherichia coli* is the predominant commensal found to be residing in the mucous layer of the mammalian colon. The bacteria and the host, by large, co-exist with mutual benefits. The pathology associated with these bacteria is the result of the immunocompromised state of the host where conditions favor the establishment of the disease. Among the pathotypes reported, the following have been well characterized, Enteropathogenic *E. coli* (EPEC), Enterohemorrhagic *E. coli* (EHEC), Enteroaggregative *E. coli* (EAEC), Enteroinvasive *E. coli* (EIEC), and Diffusely adherent *E. coli* (DAEC). Also, urinary tract infections are the most common extra-intestinal *E. coli* infection caused by the uropathopathogenic *E. coli* (UPEC).

The genes responsible for the attaching and effacing (A/E) phenotype which is the primary histopathological characteristic of EPEC infections reside in a locus called the locus of enterocyte effacement (LEE) located on a 35 kb PAI (McDaniel et al., 1995). These PAIs are typically associated with tRNA genes and possess a different GC content as compared to the genomic DNA of the bacteria. Integration of these happens at *selC* tRNA genes which have proven to be the “hot-spots for mobile gene insertions. They are also adjacent to other virulence genes, most of which code for the structural and effector proteins of the type three secretion system (T3SS) (Tauschek et al., 2002). There are five major polycistronic regions designated as LEE 1-5 comprising of 41 open reading frames (ORFs) (Kenny et al., 1997) LEE1, LEE2, LEE3 encode for T3SS apparatus that span the inner and outer membranes (Yerushalmi et al., 2014), they also code for the outer membrane porin, EscC and an ATPase called EscN. LEE4 encodes a necessary protein EspA (Knutton et al., 1998) which forms the filament structure over the secretion needle upon polymerization that is necessary for injection of effectors into the host cell, EspB and EspD which aid in pore formation in the host cell membrane (Ide et al., 2001) and EspF which is injected into the host cell that targets the mitochondria triggering the activation of cell death pathways (McNamara et al., 2001). EspF also causes redistribution of an important tight junction protein called the occludin, which results in the loss of *trans*-epithelial membrane resistance. LEE5 codes for intimin and Tir proteins and also CesT which is the Tir chaperone (Elliott et al., 1999).

The first open reading frame, LEE1 encodes for the LEE-encoded regulator (Ler) which counteracts the inhibitory effect of H-NS which is a DNA binding protein that inhibits transcription of LEE ORFs (Winardhi et al., 2014). At a temperature of 27°C, LEE1 is repressed whereas it gets activated at 37°C (Rosenshine et al., 1996). Ler protein shares amino acid sequence similarity with the H-NS (majorly the C terminal domain which binds to the DNA). Ler activates the transcription of LEE2-5 (Sperandio et al., 2000), map, the gene product is known to target mitochondria of the host cell and *espC* (Stein et al., 1996). EspC is encoded by a second PAI of EPEC which is translocated through pinocytosis and causes disruption of host cell cytoskeleton. Since Ler regulates the transcription of *espC* that does not reside in the LEE locus, it is termed as the global regulator of virulence gene expression in EPEC. In EHEC, the stcE gene on the plasmid pO157 is also regulated by Ler (Grys et al., 2005). Although it is shown that *ler* mutants are severely attenuated in their ability to form A/E lesions (Zhu et al., 2006), it is observed that *ler* is necessary only in the initial stages of infection and later represses the transcription of LEE1 (Zhu et al., 2006). Other generic *E. coli* proteins, such as, Fis, integration host factor (IHC) and BipA positively regulate LEE1 expression whereas Hha, which is a regulator of α-hemolysin directly binds to LEE1 region and negatively regulates *ler* expression (Hong et al., 2010).

Other regulatory systems like RcsC-RcsD-RcsB phosphorelay system and EHEC specific GrvA protein is known to control the expression of *ler* (Tobe et al., 2005). Transcription of the *ler* encoding LEE1 operon is found to be enhanced with the global response to starvation, entry into the stationary growth phase and is included in any form of stringent response requiring *relA* and *spoT* (Nakanishi et al., 2006).

Transcription of LEE operons, however, is not under the sole control of Ler. In EHEC, two novel regulators, YhiF and YhiE are known to regulate the secretion of EspB, EspD and Tir proteins (Tatsuno et al., 2003) which shows they regulate the transcription of LEE2 and LEE4 operons. These proteins share sequence similarity with the *luxR* family of proteins (Tatsuno et al., 2003). Also, in EHEC, two genes in the cryptic T3SS of the Sakai 813 strain, namely, *etrA* or *eivF* led to the increased secretion of EspA, EspB, Tir and also *pO157* encoded StcE and EspP (Zhang et al., 2004). Also, mitomycin C treatment is also known to induce expression of LEE2 and LEE3 in *recA* and *lexA* dependent manner (Mellies et al., 2007). LexA was found to bind to the SOS box located in between the overlapping promoter regions of LEE2 and LEE3.

The Ler transcription itself is regulated by the action of proteins encoded by the plasmid-encoded regulator (Per) locus found on a plasmid called the EPEC adherence factor (EAF) plasmid which is 70–100 kb in size and is a characteristic of typical EPEC strains (Bieber et al., 1998). The Per locus on the EAF plasmid has three regions, namely PerA, PerB and PerC (Porter et al., 2004). PerA controls the bundle Forming Pilus (BFP) expression that also helps in adhesion. PerA has amino acid sequence similarity to AraC family of proteins, which is also closely related to VirF protein of *Shigella* (Dorman and Porter, 1998). PerA also exhibits autoregulation (Ibarra et al., 2003). BFP expression is also regulated by the Cpx two-component system (TCS). This TCS comprising of the sensor kinase CpxA and response regulator CpxR is responsive to lethal damage to the cells. BFP is not assembled until this TCS is activated (Nevesinjac and Raivio, 2005). BFP also exhibits maximum expression at 37°C (Puente et al., 1996). *bfpA* gene is also under NH4^+^ ion regulation. Increased NH4^+^ is found to repress the expression of this gene (Puente et al., 1996). PerC regulates Ler expression by directly binding to the region upstream to the LEE1 leading to transcriptional repression (Kimmitt et al., 2000). In acidic conditions, the Per genes are repressed by the action of GadX which also activates GadAB genes that bring about resistance to acidic environment (Tramonti et al., 2002). This acts as a key step in regulating the virulence gene expression as the expression can only be activated once the bacteria pass through the acidic environment of the stomach and reach the alkaline environment of the small intestine.

Another protein that acts as an adhesive is a large 385 kDa protein called lymphostatin (LifA) that is encoded by the region adjacent to PAI (outside of LEE). This protein inhibits lymphocyte activation. Apart from these, the porcine A/E associated gene (paa) and Long Polar Fimbriae (LPF) also aid in adhesion. Once the expression of these virulence genes has been activated, the release of effector proteins by the T3SS into the host cells leads to the disease manifestation.

Till date, many pathotypes of *E. coli* have been identified and the list keeps growing. Additionally, with the abuse of antibiotics in the recent past, the emergence of drug-resistant bacteria is also on the rise. Thus, there is a need to better characterize the virulence gene expressions and their triggers before the number of pathotypes become overwhelming.

### Gene Regulation in *Salmonella*

*Salmonella* is a Gram-negative rod-shaped pathogen, bearing peritrichous motility and has been known to cause a pathogenicity in a wide array of hosts spanning from cold-blooded animals to humans. In humans, it causes various diseases ranging from occasionally fatal systemic fever to self-limiting diarrhea. Regardless of current medical advancement, *Salmonella* remains a major cause of morbidity and mortality in developing countries. *Salmonella* Typhimurium has a plethora of genes that are strictly regulated and thereby facilitates adaptation in various environments. There are at least 23 *Salmonella* pathogenicity islands (SPI, which are the molecular toolbox for *Salmonella* pathogenicity) in SPI pan-genome (Fookes et al., 2011; Hayward et al., 2013). In response to environmental signals such as temperature, redox state, pH, osmolarity, antimicrobial peptides and nutrient deprivation *Salmonella* achieves adaptation via well-coordinated gene regulation. The pathogenicity is caused by two steps encoded by two different T3SS, SPI-1 and SPI-2. The SPI-1 encoded T3SS acts during initial contact with the host cells, it then injects bacterial effector protein into the host cell and mediates uptake in non-phagocytic cells. To survive inside the phagosome/vacuole it then expresses SPI-2 T3SS and its effectors are then injected into the host cell. Major regulation of the gene in the bacterial domain is via transcription regulation. One prominent example is five alternative sigma factors designated ^σ^E (^σ^24), ^σ^F (^σ^28), ^σ^H (^σ^32), ^σ^S (^σ^38), and ^σ^N (^σ^54) which are activated differentially under stress or changes in growth conditions (Gruber and Gross, 2003). Upon general stress such as starvation and environmental stress response, ^σ^S upregulates approx. 500 genes (Hengge, 2009). The SPIs are AT-rich regions of the genome encoding genes required to induced phagocytosis in non-phagocytic cells (Lostroh and Lee, 2001; Patel and Galan, 2005; Fink and Cookson, 2007), negative regulator of SPI-1 is *hilE*, which has three promoters, controlled by Mlc repressor (response to glucose) (Lim et al., 2007), FimZ activator also reported to regulate *Salmonella* attachment and adherence (Baxter and Jones, 2005). Also, a key regulatory factor for SPI-1 regulation is HilD whose expression is in control of *std* fimbrial operon gene via a DNA adenine methylation pathway (Lopez-Garrido and Casadesus, 2012). In a recent report, it has been shown that in *Salmonella* the Hfq dependent sRNA Spot 42 is transcriptionally regulated by the CRP-cAMP regulatory circuit. In the same study, it was shown the upon relieving repression of Spot 42 is up-regulated *hilD* by binding to 3′UTR unstructured regions of *hilD* transcripts (El Mouali et al., 2018). Two-component systems also regulate *Salmonella* response to external environmental stimuli. One of the major two components in *Salmonella* is PhoP/Q system which functions in acid tolerance response. Classical response regulators can bind the RNA polymerase directly. Mechanism of transcriptional regulation also involves repressor DNA binding protein which remains bound to DNA and suppresses transcription unless an inducer molecule bind.

Two-component systems which is involved in combating oxidative and nitrosative stress is SoxRS, the SoxR has one (2Fe–2S) for sensing superoxide and nitric oxide radical, upon sensing it gets activated and further upregulated the expression of SoxS, which then further induces genes responsible for oxidative stress (Nunoshiba et al., 1993; Ding and Demple, 2000). The regulator of cellular NO response is NsrR, which is again an iron-sulfur cluster containing NO stress response regulator. NsrR is a transcriptional repressor and its regulon’s most conserved genes *hmp* encodes for a flavohaemoprotein which can detoxify NO in both anaerobic and aerobic conditions. It has been also reported that *hmp* expression can, in turn, exacerbate the oxidative stress by promoting Fenton Chemistry (Bang et al., 2006). The transcriptional regulator OxyR belonging to LysR family is a non-metal containing proteins reported to activate transcription via a redox and nitrosative stress sensitive Cys199 of the genes involved in oxidative stress (Kim et al., 2002; Paget and Buttner, 2003). Apart from this one more fascinating example is *Salmonella* is *mgtA* which is responsive to low magnesium. Also, a proline-rich region in the open reading frame also located within the leader mRNA of *mgtA*, named as *mgtL*. It has been demonstrated to confer responsiveness against proline and osmolarity (Park et al., 2010).

In *Salmonella*, the role of H-NS, NAP in global silencing of genes that are acquired via HGT (Lucchini et al., 2006; Navarre et al., 2006). Usually, the gene acquired has lower GC content than the resident gene, based on this difference the H-NS specifically binds to the high AT-rich region and thereby silence the acquired gene in this process (Navarre et al., 2006; Pacheco et al., 2012). Following this, the subsequent role is played by an evolutionary modification that further incorporates acquired genes into regulatory networking. To express silenced genes *Salmonella* incorporated mechanism of counter silencing such as: the antagonist binding to H-NS subsequently disrupting the multiunit complex of H-NS, competing with H-NS to bind DNA, alternative sigma factor assigned which demonstrates higher affinity for A-T rich sequence, and last is changing geometry of the promoter such that H-NS can no longer bind (Navarre et al., 2006; Stoebel et al., 2008). This type of counter silencing regulation is mostly seen in genes associated with virulence of the pathogen. One example of this kind of regulation is biofilm formation in *Salmonella*, biofilm formation helps to colonize the host and protects cells outside of the host too. In absence of H-NS the *csgBAC* genes can be even transcribed by ^σ^70 but in presence of H-NS, it requires an alternative sigma factor ^σ^S (Olsen et al., 1993). On the other hand, the master regulator *csgD* is also in turn regulated by OmpR, IHF, H-NS and MlrA by bind to upstream of the *csgD* promoter (Gerstel et al., 2003).

The SPIs are also silenced by H-NS until it is sensed by external stimuli from the host and counter silenced by a transcriptional factor. There are various transcription factors such as HilE, HilA (Lostroh and Lee, 2001). Under low osmolarity condition *hilA* is silenced until H-NS mediated the repression is relieved by two other factor HilC and HilD encoded by SPI-1 (Schechter et al., 2003).

Apart from these regulations, there is other gene regulation which is imparted by proteolytic signaling cascade and small non-coding RNA which are typically involved in the immediate gene expression/repression. One excellent example is the upregulation of σ S via σ E (Bang et al., 2005; McCullen et al., 2010) *rpoH* and *hfq*. Hfq promotes translation of ^σ^S by binding to small RNAs DsrA and RprA thereby stabilizing their interaction with *rpoS* mRNA (Sledjeski et al., 1996; McCullen et al., 2010; Soper et al., 2010). The outer membrane proteins (OMPs) also participate in sensing and gene regulation. Misfolded OMPs can activate ^σ^E (Alba and Gross, 2004; Ades, 2008), under non-stressed conditions the ^σ^E is bound to RseA an anti-sigma factor and thus remains sequestered at the inner membrane. But it has also been shown in *Salmonella* that activation of ^σ^E can be independent of misfolded OMPs. Inside acidified phagosomes of host macrophages, the low pH triggers the DegS independent activation of ^σ^E, which is essential for *Salmonella* survival (Muller et al., 2009). As a result, it was concluded that different environmental stimuli can activate common stress response regulator ^σ^E. *Salmonella* can also sense and utilize intestinal metabolites such as ethanolamine. It is sensed by EutR receptor and upregulate genes which facilitate utilization of ethanolamine during gut-inflammation scenario thereby providing it with an upper hand of survival to the commensal microbiota (Thiennimitr et al., 2011). Apart from in general gene regulation *Salmonella* maintains transcriptional regulation directly at the RNA polymerase machinery level. It has assigned different sigma factor for different environmental cues to regulate the subsequent downstream genetic locus.

### Gene Regulation in *Yersinia*

Three species of *Yersinia* are pathogenic to humans among them *Y. enterocolitica* and *Y. pseudotuberculosis*, represents gastrointestinal pathogens and it adopts the fecal-oral route of transmission (Rich et al., 1990). *Y. pestis* is a causative organism of bubonic plague. To establish infection in mammalian host successfully, it must adapt and combat multiple numbers of environmental stress from the host. Therefore, *Yersinia* regulates their gene expression in a tight fashion and only express the gene required for survival at any moment. Bacteria, in general, regulate gene expression in the level of transcription, for example, sigma factor, promoter sequence, and transcriptional activator and repressor all respond and act to environmental cues and bacterial need (Cornelis et al., 1989; Iriarte et al., 1995a,b; Trulzsch et al., 2001; Brzostek et al., 2007; Gao et al., 2008; Raczkowska et al., 2011). It is well studied that there is an intricate layer of regulation for the expression of several survival and virulence gene. One instance is the expression of Yop-Ysc type III secretion system and its effector proteins are required for virulence in mammalian system at the same time it is essential to shut off this machinery to escape host immune responses (Woestyn et al., 1994; Cornelis and Wolf-Watz, 1997; Cornelis et al., 1998; Shao, 2008). One of the excellent reviews that give enormous knowledge on transcriptional regulation in *Yersinia* is by Marceau (2005). While transcriptional control is a broader form of regulation, post-transcriptional/translational gene regulation research is on higher interest recently. The post-transcriptional research provides fine tuning of gene regulations and facilitates more rapid adaptation ability in the fleeting environment. *Yersinia* possesses various RNA binding regulatory protein which either stabilizes or degrades target mRNA thereby regulating them. *yopD* is one such effector protein a part of the T3SS translocon, in addition, it binds to 5′ untranslated region of *yopQ* (effector protein gene) mRNA along with its chaperone *lcrH* and mediates decay of the transcript (Anderson et al., 2002). The regulation of *yopQ* is also via class II regulators (yopD, *lcrH*, and Yop secretion factors *yscM1/yscM2*) which completes with the ribosome 30S and there downregulates *yopQ* translation (Kopaskie et al., 2013).

Another prominent regulator of gene expression in *Yersinia* is carbon storage regulator A (CsrA), there are various well-established mechanistic models which suggests that depending upon the binding site on the target gene, translation is either activated or repressed. CsrA binds to 5′-UTR of target mRNA and either alter translation, mRNA turnover or transcription elongation (Vakulskas et al., 2015). The function of CsrA is in turn regulated by two non-coding RNA molecules termed as CsrB and CsrC which sequester CsrA thereby regulating the amount of free CsrA present to function (Liu et al., 1997; Romeo, 1998) (Weilbacher et al., 2003; Vakulskas et al., 2015). CsrA also regulates the global transcriptional regulator RovA in *Y. pseudotuberculosis* thus controlling the expression of genes involved in virulence indirectly (Cathelyn et al., 2006) (Heroven et al., 2008). CsrA stabilizes the master regulator of flagellum biosynthesis and chemotaxis, *flhDC* by preventing cleavage of the same by cellular RNase E (Heroven et al., 2008). Recent reports suggest that CsrA also involved in fine-tuning of expression of two T3SS genetic regulatory cascades Ysa, Ysc, flagellar T3SS and biofilm formation in response to distinct environmental cues (Willias et al., 2015; Ozturk et al., 2017). The small protein SmpB functioning along with small RNA SsrA to rescue stalled ribosomes on incomplete mRNA transcripts (Komine et al., 1994; Keiler et al., 1996; Karzai et al., 2000) are also involved in regulating transcription of the master regulator gene of virulence lcrF/virF (Okan et al., 2006). Apart from this SsrA is important for *Y. pestis* virulence via intranasal and intravenous routes and ΔssrA strains were also used as candidate vaccine in the same study (Okan et al., 2010). Another protein which plays a central part in the regulation of a large number of genes as much as 243 genes in *Yersinia* (Geng et al., 2009) is a pleiotropic small RNA chaperone protein Hfq. The Hfq is 11 KDa in *Yersinia*, forms homo-hexameric ring complex that allows it to bind more than one RNA molecule at any given point (Kajitani and Ishihama, 1991; Vytvytska et al., 1998; Sauter et al., 2003). The site of binding of Hfq to small RNA and target mRNA determines whether the transcript will be stabilized or degraded (Vytvytska et al., 1998; Masse et al., 2003b; Meibom et al., 2009). The Hfq regulates genes involved in virulence, metabolism (carbohydrates, nitrogen, iron, fatty acids, and ATP), siderophore (Kakoschke et al., 2014) and surface pathogenicity factor, and stress response (Kakoschke et al., 2016). Some of the genes regulate are plasminogen activator protease gene *pla*, the F1 antigen gene *cafI*, the diguanylate cyclase gene *hmsT* and 50% gene of T3SS. Hfq also participates in the repression of non-flagellar dependent swarming motility (Geng et al., 2009) and biofilm formation *in vitro* (Kakoschke et al., 2014). On the other hand, it is also required for the formation of biofilm inside proventriculus of flea thereby maintaining the life cycle in flea vector (Rempe et al., 2012). Hfq deficient strains showed growth impairment, higher sensitive to environmental stresses and antibiotics, along with disability in QS, and host invasion. One of the recent reports suggests that various Hfq-dependent phenotypic alteration in *Y. enterocolitica* is mediated by depression of the transcriptional regulator RovM (master regulator of motility, chemotaxis and flagella synthesis) (Leskinen et al., 2017).

A different set of elegant regulation is mediated by small RNAs in *Yersinia*, majorly regulating genes which are required to express/repress immediately upon a change in environment. In response to temperature shift from 26 to 37°C (host temperature), there is differential sRNA expression in *Y. pestis* which enhances host infection (Beauregard et al., 2013; Schiano et al., 2014), it was also reported in infected mice’s lungs (Yan et al., 2013). One of the candidates sRNAs, ysr35, deletion resulted in attenuation of *Y. pestis* in mice models (Koo et al., 2011). There are small RNAs such as *ysr141* expressed on *pCD1* plasmid that encodes T3SS effectors injected into the host system to escape host immune system and establish infection successfully (Schiano et al., 2014). Ysr141 targets 5′ UTR of *yopJ* (T3SS effector) and activates its synthesis. Also, in *Y. pestis* sRNA HmsB has been shown to upregulate biofilm formation (Fang et al., 2014). One report suggests that upon knocking-down of *ysr170* there was a significant decrease in infection efficiency of pathogenic *Yersinia* resulting in higher production of the proinflammatory cytokines TNF-alpha, IL-8 and EGR1. In the same study, *ysr170* was proposed to the global regulator of virulence and metabolism in *Yersinia* (Li et al., 2016). Another mode of regulation in *Yersinia* involves RNase E mediated degradation along with Hfq of cellular mRNA of T3SS effector YopE (Yang et al., 2008) in post-translational step, similar regulations have also been reported for PNPase (Rosenzweig et al., 2005; Rosenzweig and Schesser, 2007). There is moreover a tight regulation maintained during the change in surrounding temperature. A report which states that over 400 genes are differentially regulated at two different temperature 26 and 37°C. Out of which 61% of them are downregulated, whereas 39% were upregulated (Han et al., 2004). Many of these genes are associated with T3SS which gets activated exclusively inside mammalian host at 37°C by de-repressing the transcription of *lcrF*, encoding for AraC like transcription factor. Therefore, it has been proposed that *lcrF* is thermo-sensor and mutation of the same, critically hampers the virulence of the pathogen (Hoe and Goguen, 1993). The secondary structure of *lcrF* also forms motif homologous to heat shock protein in eubacteria (Kortmann and Narberhaus, 2012; Schumann, 2012). Regarding riboswitches the mechanism underlying is yet to be discovered, prediction of one of the bioinformatics studies show there are a few switches like FMN, cobalamin, *yybP-ykoY* element, Moco which are yet to be characterized. One of the well-characterized riboswitches is *mgtA*, which is a Mg^2+^ responsive (Korth and Sigel, 2012). Among all the enteropathogens, most diverse set of gene regulation is shown by *Yersinia.* There is a growing need for more research in this field as this would lead us to some very important clues to how these deadly pathogens regulate their genetic locus to effectively establish the infection with high rates of mortality and morbidity. Genetic regulations in *Yersinia* also gives us insights that not only it invade the host system but there are specific set of regulations performed which mask them from host immune system and thereby escaping the same.

### Gene Regulation in *Shigella*

*Shigella* causes bacterial dysentery in humans. The disease progresses in the lower part of the digestive tract. The bacteria invade colonic epithelial cells, reproduces within them and proceeds to other cells. The genes necessary for *Shigella* virulence are present on a 230-kbp plasmid and these are clustered in a 31 kbp portion (Dorman and Porter, 1998). *S. flexneri* virulence genes belong to the large family of H-NS repressed genes (Dorman and Porter, 1998). In addition to H-NS and StpA, *Shigella* *flexneri* 2a encodes Sfh, a third member of H-NS like family (Gore and Payne, 2010).

CsrA and Cra have antagonist effects on glycolysis and gluconeogenesis and it affects epithelial cell invasion in early stages (Gore and Payne, 2010). *pfkA* is a gene required for glycolysis. CsrA positively regulates *pfkA* expression (Sabnis et al., 1995) while Cra negatively regulates the same (Chin et al., 1989). Gore and Payne (2010) showed that expression of virulence gene regulators *virF* and *virB* were reduced in *pfkA* and *csrA* mutants (Gore and Payne, 2010).

The VirF-VirB modulatory mechanism controls the expression of *ipa* genes. This regulation of the expression of *ipa* (invasion plasmid antigens) genes is subject to environmental factors like osmolarity, temperature and pH (Bernardini et al., 1990; Porter and Dorman, 1997a; Jennison and Verma, 2004). Transcription of the virulence genes on the plasmid has shown to require *virF* and *virB* genes (Adler et al., 1989).

VirF is a member of AraC family of transcription regulators that exerts influence on gene expression usually in response to temperature (Gallegos et al., 1997). IHF is necessary for the complete activation of *virF* and *virB* (Porter and Dorman, 1997a) while H-NS represses expression of *virB* (Tobe et al., 1993). Transcription of the *virF* gene is under pH control. But thermal regulation can supersede pH control at genes downstream in the regulatory cascade (Porter and Dorman, 1997a,b). In *S. sonnei*, and almost certainly also in *S.* *flexneri*, this pH control depends on the *cpxRA* gene located on the chromosome. *cpxR*, the gene encoding the response regulator, binds at *virF* and regulates it in response to pH (Nakayama and Watanabe, 1995).

The Hfq protein is an RNA binding protein. It performs several physiological roles in bacteria (Hayashi-Nishino et al., 2012; Kakoschke et al., 2014; Vogt and Raivio, 2014). Hfq accommodates for environmental stress and affects virulence in S. *flexneri* (Yang et al., 2015). Hfq augments acid resistance in *S. flexneri* by regulating acid resistance genes-*gadB*, *gadA*, *hdeB*, *hdeA*, and *hdeD*. Hfq on top plays a significant role in the virulence of *Shigella* by positively regulating T3SS gene expression (Yang et al., 2015).

In *Shigella*, when the iron is replete, the transcription factor Fur binds to iron and Fe-Fur restricts the expression of iron transport genes (De Lorenzo et al., 1988). *ryhB* encodes a small RNA that promotes degradation of transcripts for iron storage, oxidative metabolism and stress proteins and Fe-Fur represses expression of *rhyB* too (Masse et al., 2003a, 2005) Iron availability modulates both transcription of fur and the activity of the Fur protein (De Lorenzo et al., 1988; Delany et al., 2002; Sala et al., 2003; Hernandez et al., 2006). ArcA represses expression of fur and it is equally involved in the regulation of *feo* and siderophore biosynthesis and transport genes (Boulette and Payne, 2007). ArcA (aerobic respiration control A) is a global transcription factor and *feo* is an iron transport system encoded by *S. flexneri* which transports ferrous ion. Oxygen availability also plays an important role as a general signal for regulation of iron acquisition (Boulette and Payne, 2007).

SlyA in *S. flexneri* is involved in the regulation of virulence-associated genes and in acid resistance. In *S. flexneri*, the *slyA* promoter activity is most during the stationary phase (Weatherspoon-Griffin and Wing, 2016). *slyA* promoter is negatively autoregulated and positively regulated by PhoP (Norte et al., 2003; Santiago et al., 2016).

The *Shigella* virulence gene regulon is an example of a complex gene control system. However, a detailed characterization of the gene regulatory mechanisms needs to be discovered.

## Conclusion and Future Direction

In the review, we have tried to summarize some of the common as well as pathogen specific gene regulatory mechanisms employed by enteropathogenic bacteria. Since the amount of literature available with respect to gene regulation in these bacteria is huge, we have tried to include some of the major and elegantly regulated mechanisms. For example, in QS, AI 1, AI 2, AI 3 mediated genetic control, different types of TA systems induced gene regulation and other direct transcription regulators like NAPs. Emerging literature on NAPs are suggesting that there might be a very thin line between the conventional transcriptional regulators and NAPs. We have also summarized the pathogen specific genetic regulation in *E. coli, Salmonella* sp.*, Yersinia* sp*., Shigella* sp. which majorly comprise the class of entero-pathogenic bacteria.

Recently, with the advent of bioinformatics, studying gene regulation has become far simpler. Prediction, detection and designation of functions of virulence-associated genes (like TA systems) have become a matter of few hours. Since the rate of failure of antibiotics is increasing with the emergence of multiple drug resistance strains, we are in dire need of novel strategies to target these pathogens. Studies on NAPs and other novel proteins that directly regulate the survival of bacteria are on the rise, which might prove to be successful in being targeted for therapeutics. Also, the design of small molecule inhibitors of PAMPs, nanoparticle-based drug delivery systems has been proven to be successful. Although these pathogens have been associated with causing foodborne illness since decades, our failure in being able to control them is the proof that there is a gap to be filled in the knowledge of our understanding their survival strategies. It should also be considered that the need of therapeutics is majorly in economically backward countries in Africa and south-eastern Asia and thus the novel strategies to control disease should be feasible for use in these countries for bringing down the numbers in the statistics of morbidity and mortality associated with such illnesses.

## Author Contributions

RC, MS, RS, and DC designed and prepared the manuscript. DC supervised the work and all the authors were involved in the final editing of the manuscript.

## Conflict of Interest Statement

The authors declare that the research was conducted in the absence of any commercial or financial relationships that could be construed as a potential conflict of interest.
